# Cholecalciferol Inhibits Cell Growth and Induces Apoptosis in the CaSki Cell Line

**DOI:** 10.3390/medsci8010012

**Published:** 2020-02-13

**Authors:** Sachin Bhoora, Yuvelia Pather, Sumari Marais, Rivak Punchoo

**Affiliations:** 1 Department of Chemical Pathology, Faculty of Health Sciences, University of Pretoria, Pretoria 0083, South Africa; 2 Department of Physiology, Faculty of Health Sciences, University of Pretoria, Pretoria 0083, South Africa; 3 National Health Laboratory Services, Tshwane Academic Division, Pretoria 0083, South Africa

**Keywords:** cholecalciferol, apoptosis, CaSki, cervical cancer, vitamin D, cell viability

## Abstract

Vitamin D has displayed anti-cancer actions in numerous in vitro studies. Here, we investigated the anti-cancer actions of cholecalciferol, a vitamin D precursor, on a metastatic cervical cancer cell line, namely, CaSki. Experimental cultures were incubated for 72 h and treated with cholecalciferol (10–1000 ng/mL). In the present study, cell count, viability, proliferation and cell cycle were analyzed by a crystal violet assay, trypan blue assay, Ki67 proliferation, and a cell cycle assay, respectively. Biomarkers of apoptosis, necrosis, and autophagic cell death were measured by the Caspase 3/7 and Annexin V/7-AAD Muse™ assays, a LC3-II assay, and a lactate dehydrogenase release assay, respectively. The ultrastructural features of cell death were assessed by transmission electron microscopy. A statistical analysis was performed using a one-way ANOVA and Bonferroni’s post-hoc analysis test, and *p* < 0.05 is considered statistically significant here. The results identify statistical decreases in cell count and viability at high-dose treatments (100 and 1000 ng/mL). In addition, significant increases in apoptotic biochemical markers and apoptotic ultrastructure are shown to be present at high-dose treatments. In conclusion, high-dose cholecalciferol treatments inhibit cell count and viability, which are both mediated by apoptotic induction in the CaSki cell line.

## 1. Introduction

The anticancer activity of vitamin D has been demonstrated in various cancer cell lines [1]. Clinical data have also demonstrated that vitamin D has anticancer activity against prostate, colon, and breast cancers [1,2].

Vitamin D precursors are systemically activated into biologically active hormones by two sequential hydroxylation reactions. Cholecalciferol is activated by conversion to 25-hydroxycholecalciferol (calcidiol) through vitamin D 25-hydroxylases (CYP2R1 and CYP27A1) in the liver, and 25-hydroxycholecalciferol is subsequently activated by conversion to 1,25-dihydroxycholecalciferol (calcitriol) through 1-α hydroxylase (CYP27B1) in kidneys. Systemic vitamin D homeostasis is maintained by 24-hydroxylase (CYP24A1), which catabolizes calcidiol and calcitriol into inactive excretory metabolites [3]. Calcitriol binds intracellular vitamin D receptors (VDRs), which then heterodimerize with the retinoid X receptor (RXR) and co-activators. The complex then binds to promoter regions of vitamin D response elements, which modulate the expression of various genes for skeletal and nonskeletal homeostasis [3,4].

Some tissues, such as cervical tissue, possess a vitamin D metabolizing system (VDMS). This system mimics systemic vitamin D metabolism and consists of similar intracellular vitamin D activating and inactivating enzymes, as well as VDRs [5,6,7]. The VDMS regulates cell growth, differentiation, and cellular metabolism. Vitamin D deficiency states, therefore, are implicated in tumorigenesis [4,8].

The prevalence, morbidity, and mortality of cervical cancer are all high in developing countries, and primary and secondary medical interventions—including nutritional support—are needed. Although an autocrine VDMS has been identified in cervical tissue [5,7], there are limited studies that have explored the anticancer effect of vitamin D in cervical cancer.

The aim of this investigation was to determine the action of cholecalciferol, a vitamin D precursor, on cell growth and cell death on the cervical metastatic cancer cell line CaSki. The results show statistically significant decreases in cell count and viability and significant increases in the proportion of cells in the sub-G_1_ fraction as well as the rate of apoptosis in the CaSki cell line at high-dose cholecalciferol treatments (100 and 1000 ng/mL) in comparison to both control cultures and low-dose cholecalciferol treatments (10 and 40 ng/mL).

## 2. Materials and Methods

### 2.1. Cell Culture and Treatments

The CaSki cell line (CRL-1550™) was purchased from the American Type Culture Collection (ATCC) and grown in Dulbecco’s Modified Eagle’s Medium (DMEM) (Invitrogen, Carlsbad, CA, USA). The medium was supplemented with 10% fetal calf serum, streptomycin (100 μg/mL), penicillin G (100 U/mL), and fungizone (250 μg/L) (Sigma-Aldrich, St. Louis, MO, USA). Cell cultures were incubated in a humidified temperature-regulated incubator at 37 °C and a 5% CO_2_ atmosphere (Waltham, MA, USA).

Exponentially growing CaSki control and experimental cultures were incubated for 72 h at 37 °C and a 5% CO_2_ atmosphere. Cells were seeded at a density of 25,000 cells/mL for cell count and viability assays, 50,000 cells/mL for flow cytometry, and 100,000 cells/mL for electron microscopy, and a 16-h cell attachment protocol was used before treatments. CaSki experimental cultures were exposed to a range of cholecalciferol treatments (10, 40, 100, and 1000 ng/mL), which were prepared from cholecalciferol (Sigma-Aldrich) dissolved in analytical grade ethanol (Sigma-Aldrich). Additionally, DMEM medium-only and ethanol solvent controls were included in each experiment. The solvent control was treated with diluent ethanol diluted with the culture medium to a concentration corresponding to the 1000 ng/mL cholecalciferol treatment and to a final concentration of 0.1% (*v/v*) ethanol. The cells were washed with 1× phosphate-buffered saline (PBS) [9] and passaged using trypsin (0.25%)–ethylenediaminetetraacetic acid (0.02%) (Sigma-Aldrich).

### 2.2. Crystal Violet Cell Count Assay

During cell death, adherent cells detach from cell culture plates. By staining cells attached to the plate, the stained nuclei and proteins within the cells which bind crystal violet can be enumerated after cholecalciferol treatment [10]. The crystal violet assay, described by Feoktistova et al., was used to enumerate cells in the control and experimental cultures [10]. Briefly, cells were fixed with 1% glutaraldehyde for 15 min at room temperature. The fixative was discarded and 0.1% crystal violet solution was added to each well and then incubated at room temperature for 30 min. After incubation, the 96-well plates were first washed for 5 min by submerging the plate under tap water and then left to dry overnight. A 200 µL aliquot of 0.2% Triton X-100 was added to each well and incubated for 30 min at room temperature to solubilize the cells. After incubation, 100 µL of the solution was added to a clean, flat-bottom 96-well plate. The ELx800 Universal Microplate Reader (Bio-Tek Instruments Inc., Winooski, VT, USA) was used to read the absorbance of the samples at 570 nm. Absolute cell count was corrected for blank absorbance, and cell counts for the medium and experimental cultures were expressed as a percentage of the solvent control.

### 2.3. Cell Viability

The trypan blue dye exclusion assay was used to determine the number of viable cells [11]. Cells undergoing cell death have compromised cell membranes and therefore, take up trypan blue dye, which is conversely excluded by live cells [11]. Briefly, 20 μL of the cell suspension from each control and treated sample was added to 80 μL of 1× PBS (1:4 ratio), and then further diluted at a 1:1 ratio with 20 μL trypan blue dye. This solution was loaded onto an Improved Neubauer hemocytometer (RS 748, depth 0.1 mm, 1/400 mm^2^, Weber Scientific, Hamilton, NJ, USA) and examined immediately under a 10× magnification using a Zeiss Axiovert-40 light microscope (Zeiss, Oberkochen, Germany).

To quantify the viable cells, the following formula [11] was applied: *Viable cells* (%) = [1 − (*Number of blue cells* ÷ *Number of total cells*)] × 100.

### 2.4. Cell Proliferation

The Ki67 nuclear antigen is only expressed in cells undergoing active cell division and is a reliable marker for cell proliferation [12]. The proliferation of CaSki cultures was determined using the Muse™ Ki67 Proliferation Kit (Merck Inc., Darmstadt, Germany). The manufacturer’s instructions were followed in the harvesting, incubation, and staining of cultures. The Muse™ Cell Analyzer enumerated the percentages of Ki67-positive cell populations in the control and experimental cultures [13].

### 2.5. Cell Cycle Analysis

The cell cycle is a fundamental process in eukaryotic cells and results in cell growth and division into two identical daughter cells. A dysregulated cell cycle is characteristic of most cancers, due to mutations in cell cycle regulatory proteins [14]. The cell cycle progression of the CaSki cell line was analyzed by quantification of the DNA content using the Muse™ Cell Cycle Assay [15] and following the manufacturer’s instructions for the harvesting, fixing, and staining of cell cultures. Briefly, CaSki cell cultures were harvested and resuspended in 1× PBS using a vortex, and fixation was carried out by adding ice-cold 70% ethanol in a dropwise manner prior to storage for 24 h at 4 °C. Thereafter, the Muse™ Cell Cycle Reagent, which contains ribonuclease and propidium iodide, was added to the fixed cells, and the DNA content was analyzed with the Muse^®^ Cell Analyzer. The proportion of cells in each phase of the cell cycle was expressed as a percentage.

### 2.6. Detection of Early and Late Markers of Biochemical Apoptosis

Apoptosis is a process of programmed cell death, characterized by distinct biochemical, nuclear, and morphological characteristics, which is essential for normal cell turnover. During the early stages of biochemical apoptosis, cells externalize phosphatidylserine to the outer surface of the cell membrane. The Muse™ Annexin V detection kit was used to detect early apoptosis by determining the binding of Annexin V to externalized phosphatidylserine on the cell membrane surface [16]. Furthermore, the 7-aminoactinomycin-D (7-AAD) necrosis marker, which binds double-stranded DNA, indicates compromised cell membrane integrity. The manufacturer’s instructions were followed in the preparation of samples, which briefly included staining the cells with Muse™ Annexin V and the dead cell reagent for 20 min in the dark before analysis with Muse™ Cell Analyzer, which quantified the percentages of live, apoptotic, and dead cells in control and experimental cultures [17].

Pro-apoptotic signals activate initiator caspases (cysteine-dependent aspartyl-specific proteases), which cause the activation of the caspase cascade and the breakdown of structural intracellular proteins. Caspase-3 and caspase-7 affect terminal events of the biochemical apoptotic pathway [16]. The Muse™ Caspase-3/7 Kit was used to measure caspase-3 and caspase-7 activation. This assay simultaneously determines the count and percentage of cells in various stages of apoptosis based on the activity of caspase-3/7 activity in combination with a dead cell dye (7-AAD). The dead cell dye indicates cell membrane structural integrity and cell death as it is excluded from live, healthy cells and early apoptotic cells. Briefly, the Muse™ Caspase-3/7 Reagent was added to cell suspensions and incubated for 30 min in the dark at 37 °C in a 5% CO_2_ atmosphere. Then, the Muse™ Caspase 7-AAD working solution was added and incubated at room temperature, protected from light, for 5 min. Cells were analyzed using the Muse™ Cell Analyzer and expressed as percentages of healthy, apoptotic, and dead cells in the control and experimental cultures [18].

### 2.7. Autophagic Cell Death

Autophagic cell death is a catabolic process that is associated with diverse diseases, including cancer. During autophagy, cytoplasmic materials and organelles are engulfed by a double membrane structure, where the autophagosome is then delivered to the lysosome for degradation. An essential protein in autophagosome elongation is the microtubule-associated protein 1A/1B light chain 3B (LC3-II), which provides an indication of the presence of autophagosomes [19]. The Muse™ LC3-II detection kit was used to determine autophagic cell death due to cholecalciferol by quantifying LC3-II expression. The cells were harvested with trypsin and incubated with the anti-LC3 AlexaFluor^®^ 555-conjugated antibody for 30 min on ice and protected from light. The tubes were then centrifuged at 300× *g* for 5 min at 4 °C and then washed with the 1× assay buffer. Finally, the cells were resuspended in the 1× assay buffer and analyzed with the Muse™ Cell Analyzer (Merck, Darmstadt, Germany), which quantified cells expressing LC3-II positivity as the mean autophagic intensity [19].

### 2.8. Necrotic Cell Death

Necrotic cell death results in the loss of membrane integrity, swollen intracellular organelles, and adenosine triphosphate (ATP) depletion [20]. Necrotic cell death, therefore, is accompanied by the release of intracellular lactate dehydrogenase (LDH) into the surrounding culture medium.

LDH was quantified using a manual LDH assay [20]. The maximum release wells consisted of cells exposed to 2% Triton X-100 for 45 min in a temperature-regulated incubator (37 °C and 5% CO_2_ atmosphere). Following incubation of the control, experimental, and maximum release cultures, the microplate was centrifuged at 100× *g* for 10 min. Then, 100 μL of the supernatant from all wells was transferred to a new 96-well plate, after which 50 μL of the reconstituted 2× LDH assay buffer was added to the supernatant and then incubated for 30 min at room temperature, protected from light. Afterwards, 50 μL of acetic acid was added to each well and thoroughly mixed. The absorbance of each control (untreated well) and treatment well (test well) was measured at 490 nm using the ELx800 Universal Microplate Reader (Bio-Tek Instruments Inc., Winooski, VT, USA). Absorbance readings were corrected against the blank, and the percentage of cytotoxicity was calculated using the formula from [20]:

$$\frac{\text{Corrected reading from solvent control or experiment wells}-\text{Corrected reading from medium only well}}{\text{Corrected reading of maximum LDH release control}-\text{Corrected reading from medium only well}}\times 100$$

### 2.9. Morphological Analysis

The ultrastructural features of classical modes of cell death were analyzed by transmission electron microscopy (TEM). Standardized protocols were used for embedding and staining the control and experimental cultures, and were performed on three biological repeats [21,22]. Briefly, cells were harvested using trypsin, fixed with 2.5% glutaraldehyde in a 0.075 M phosphate buffer for 1 h, stained with osmium tetroxide for 1 h, dehydrated with a graded series of ethanol, and then embedded in Embed 812 resin. Ultra-thin sections (80–100 nm) were prepared using a microtome and mounted onto copper grids contrasted with 4% aqueous uranyl acetate for 10 min and Reynold’s lead citrate for 10 min. The samples were viewed and photographed using the JOEL JEM 2100F (JOEL Ltd., Tokyo, Japan) transmission electron microscope and analyzed qualitatively for ultrastructural features of apoptosis, necrosis, and autophagic cell death [23,24,25,26].

### 2.10. Statistical Analyses

All experiments were conducted in triplicate using three independent biological experiments. The results are expressed as means ± standard errors of the mean (SEM). The statistical analyses were performed using a one-way analysis of variance (ANOVA) and Bonferroni’s post hoc test via GraphPad Prism version 7.0 (La Jolla, CA, USA), and *p* < 0.05 was considered statistically significant.

### 2.11. Ethics Approval

Ethics approval was obtained from the Research Ethics Committee, Faculty of Health Sciences, University of Pretoria, Pretoria, South Africa (ethics reference number: 379/2017).

## 3. Results

### 3.1. Cholecalciferol Inhibited Cell Count and Viability at High-Dose Treatments

**Figure 1:**
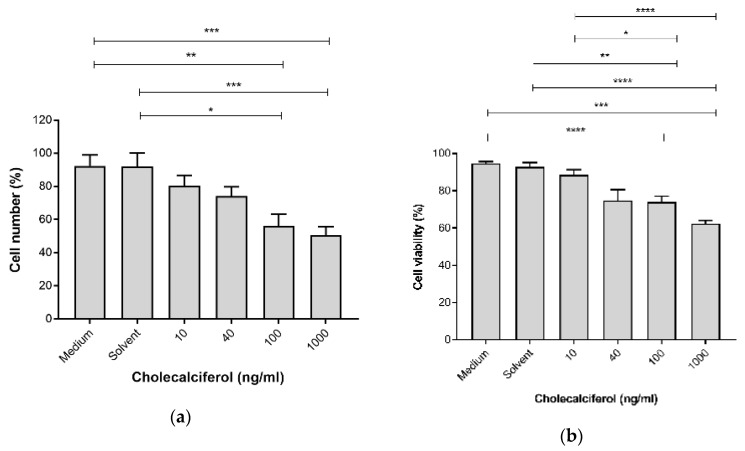
Cell count (**a**) and cell viability (**b**) in the control and experimental CaSki cultures. Cholecalciferol caused statistical inhibition at 100 and 1000 ng/mL treatments in comparison to the low-dose treatment (10 ng/mL) and control cultures. Experiments were performed in triplicate on three biological repeats. Values are expressed as mean ± SEM (*p* < 0.05 was considered statistically significant). * *p* < 0.05; ** *p* < 0.01; *** *p* < 0.001; **** *p* < 0.0001.

The cell growth parameters of cell count and viability were assessed to determine the inhibitory action of cholecalciferol treatments on the cell number and cell viability using the crystal violet assay and the trypan blue dye exclusion assay, respectively. Cell count (Figure 1a) was significantly decreased at 100 ng/mL (56.25% ± 7.08%) and 1000 ng/mL (50.59% ± 5.14%) cholecalciferol in comparison to the medium (92.40% ± 6.62%) and solvent (91.98% ± 8.31%) controls. Cell viability (Figure 1b) significantly decreased at 100 ng/mL (73.94% ± 3.16%) and 1000 ng/mL (62.41 ± 1.64%) cholecalciferol in comparison to the medium (94.71% ± 1.05%), solvent l (92.95% ± 2.13%) controls as well as 10 ng/mL treatment (88.47% ± 2.75%).

**Figure 2:**
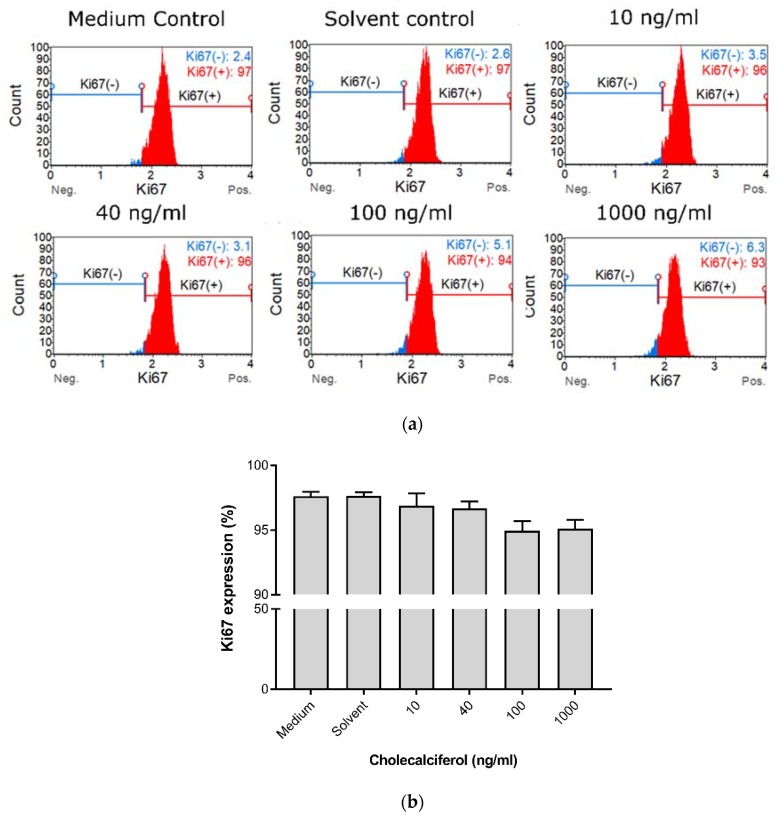
Cell proliferation measured by Ki67 nuclear antigen expression in the cholecalciferol control and experimental CaSki cultures. (**a**) Histogram of Ki67 expression in the control and experimental CaSki cultures. (**b**) Ki67 expression in the control and experimental CaSki cultures. Cholecalciferol did not cause statistical changes in cell proliferation between the control and experimental cultures. The analysis was performed in triplicate on three biological repeats. Values are expressed as mean ± SEM (*p* < 0.05 was considered statistically significant).

Cell proliferation was measured to characterize the effect of cholecalciferol on the ability of cells to actively divide as measured by the Ki67 Muse™ assay (Figure 2). Insignificant statistical changes in cell proliferation between controls and experimental cultures were observed, however, a dose-dependent decrease in cell proliferation was observed in the experimental cultures.

Collectively, these growth experiments demonstrate that treatment of CaSki with 100 and 1000 ng/mL cholecalciferol led to reduced cell count and viability in comparison to treatment with a lower dose (10 ng/mL) and the controls, however, there was no significant decrease in the cell proliferation index.

### 3.2. Cell Cycle Analysis Demonstrates Increased Sub-G_1_ Phase at High-Dose Treatments

**Figure 3:**
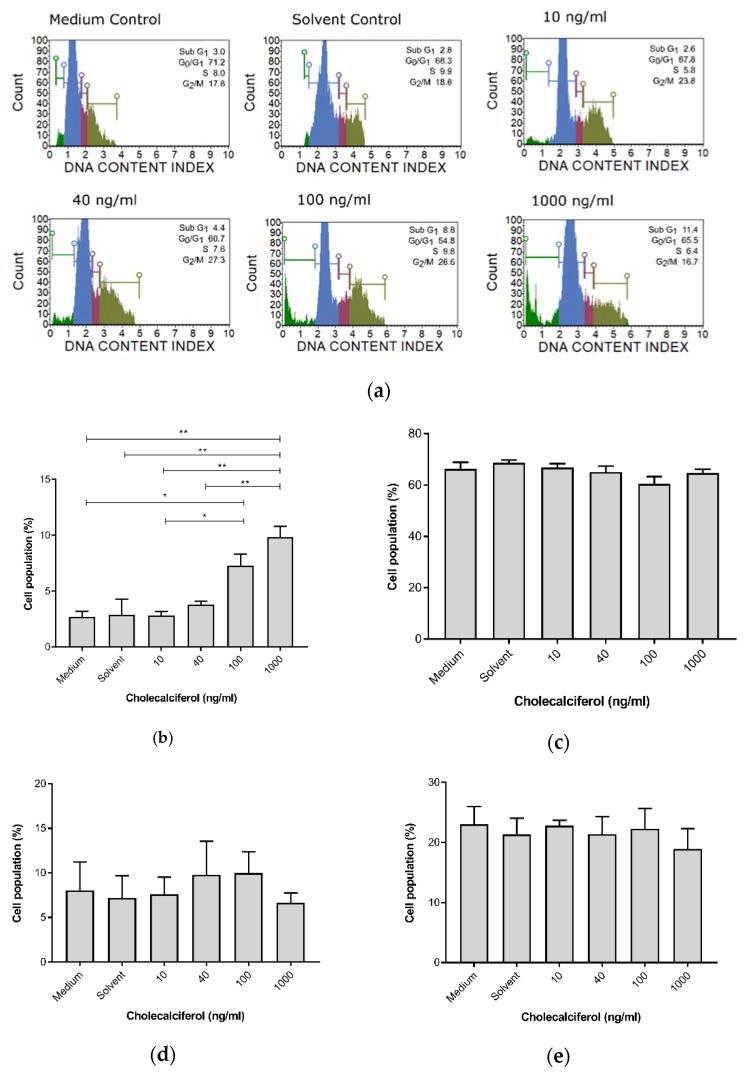
Cell cycle analysis of DNA content by the Muse™ Cell Cycle assay in the CaSki, control, and experimental cultures. (**a**) DNA distribution histogram of the CaSki, control, and experimental cultures at different phases of the cell cycle. (**b**) Cells in the sub-G_1_ phase, (**c**) G_1_/G_0_ phase, (**d**) S phase, and (**e**) G_2_/M phase. Cholecalciferol treatment significantly increased the sub-G_1_ phases at the 100 and 1000 ng/mL treatments in comparison to the low-dose treatments and control cultures. Values are expressed as mean ± SEM (*p* < 0.05 was considered statistically significant). * *p* < 0.05; ** *p* < 0.01.

For cell cycle analyses, the DNA content of cells at different stages of the cell cycle was assessed and determined using the Muse™ Cell Cycle assay. Cholecalciferol treatment significantly increased the sub-G_1_ cell population, which is consistent with cell death at 100 ng/mL (7.30% ± 1.04%) and 1000 ng/mL (9.75% ± 1.65%) in comparison to the medium (2.7% ± 0.51%) and solvent (2.9% ± 1.4%) controls, as well as the low-dose cholecalciferol treatments at 10 ng/mL (2.83% ± 0.34%) and 40 ng/mL (3.80% ± 0.31%). No statistically significant changes to cell populations were identified in the G_1_/G_0_, S, and G_2_/M phases of the cell cycle (Figure 3).

### 3.3. Cholecalciferol Induced Biochemical Apoptosis in CaSki Cell Line

The potential for cholecalciferol to induce biochemical apoptosis was analyzed using the Muse™ Annexin V detection kit for the detection of externalized phosphatidylserine residues on the cell membrane in early apoptosis, and the Muse™ Caspase 3/7 detection kit was used for identification of executioner caspase-3 and -7 activation in late apoptosis. The Muse™ LC3-II detection kit analyzed autophagic cell death, and the LDH assay measured necrotic cell death.

**Figure 4:**
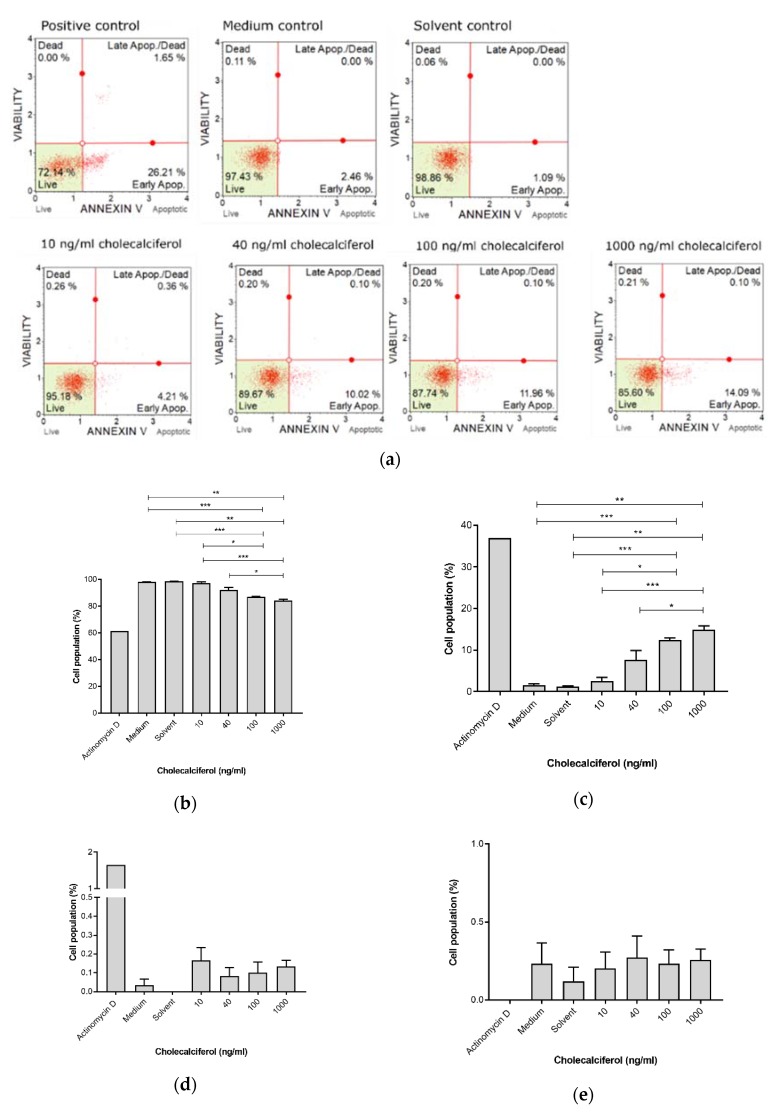
Early biochemical apoptosis in the control and experimental CaSki cultures, using the Muse™ Annexin V assay. (**a**) Two-dimensional dot-plot diagrams, and cell populations. (**b**) Live cells. (**c**) Cells in early apoptosis. (**d**) Cells in late apoptosis and (**e**) dead cells. Cholecalciferol significantly decreased the live cell population and increased the proportion of cells in early apoptosis at 100 and 1000 ng/mL in comparison to the low-dose treatments and control cultures. Assays were performed on three biological replicates, and each replicate was technically triplicated. Values are expressed as mean ± SEM (*p* < 0.05 was considered statistically significant). * *p* < 0.05; ** *p* < 0.01; *** *p* < 0.001.

The Muse™ Annexin V detection assay (Figure 4) revealed a significant decrease in live cells at 1000 ng/mL (83.99% ± 1.01%) and 100 ng/mL (87.00% ± 0.25%), in comparison to the medium control (98.03% ± 0.15%), solvent control (98.6% ± 0.15%), and 10 ng/mL treatment (97.07% ± 0.96%). Additionally, there was a significant decrease in the live cell population when treated with 1000 ng/mL cholecalciferol in comparison with 40 ng/mL (91.97% ± 2.11%).

Early apoptosis was significantly increased at 100 ng/mL (12.47% ± 0.47%) and 1000 ng/mL (14.86% ± 0.97%) cholecalciferol in comparison to the medium (1.60% ± 0.25%) and solvent (1.20% ± 0.21%) controls as well as treatment with 10 ng/mL (2.56% ± 0.90%). Additionally, there was significant increase in early apoptotic cell population at 1000 ng/mL in comparison to treatment with 40 ng/mL (7.67% ± 2.23%).

**Figure 5:**
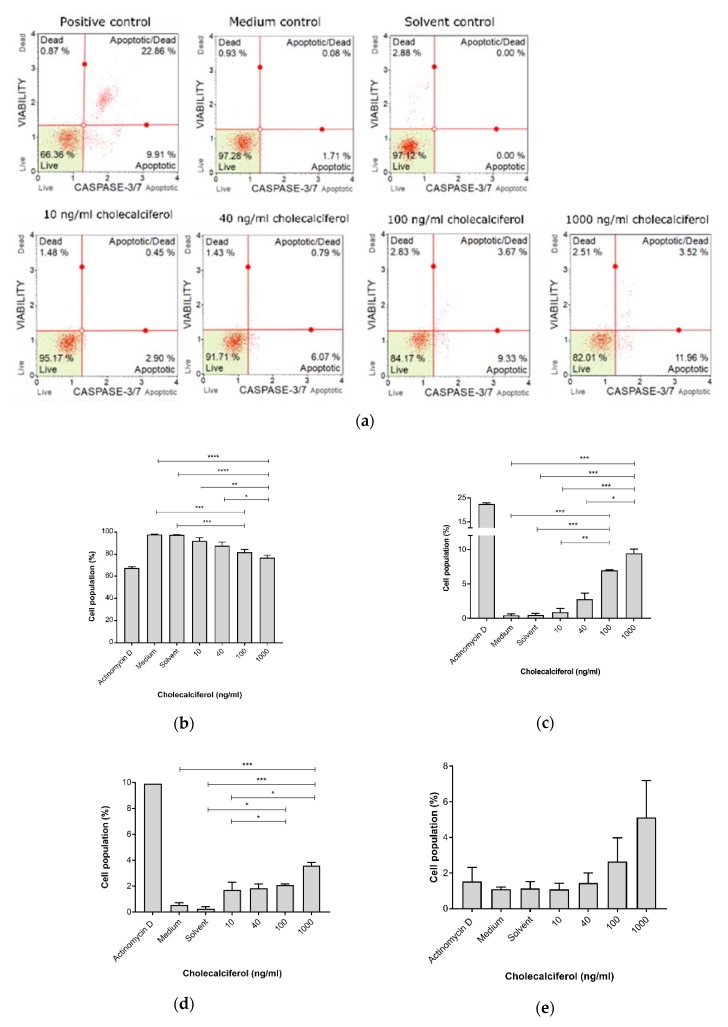
Caspase-3 and caspase-7 activation, measured by the Muse™ Caspase 3/7 detection kit. (**a**) Two-dimensional dot-plot diagrams. (**b**) Live cells, (**c**) cells undergoing apoptosis, (**d**) cells in late apoptosis, and (**e**) dead cells. Cholecalciferol significantly decreased the live cell population and apoptotic cells at 1000 ng/mL in comparison to the low-dose treatments and control cultures. Assays were performed on three biological replicates, and each replicate was technically triplicated. Values are expressed as mean ± SEM (*p* < 0.05 was considered statistically significant). * *p* < 0.05; ** *p* < 0.01; *** *p* < 0.001; **** *p* < 0.0001.

Late biochemical apoptosis was measured using the Muse™ Caspase 3/7 detection kit (Figure 5). The caspase-3 and caspase-7 assays identified significant decreases in live cell populations (Figure 5b) at 100 ng/mL (81.85% ± 2.24%) and 1000 ng/mL (76.88 ± 2.18%) in comparison to the medium (97.74% ± 0.26%) and solvent (97.26% ± 0.47%) controls and the 10 ng/mL treatment dose (91.87% ± 2.60%). Additionally, significant increases in the apoptotic cell population (Figure 5c) were observed for treatment with 100 ng/mL (6.94% ± 0.10%) and 1000 ng/mL (9.43% ± 0.62%) cholecalciferol in comparison to medium (0.45% ± 0.19%) and solvent (0.46% ± 0.26%) controls and the 10 ng/mL treatment dose (1.71% ± 0.60%). Furthermore, significant increases in apoptotic/dead cell populations (Figure 5d) were observed at 100 ng/mL (2.08% ± 0.10%) and 1000 ng/mL (3.59% ± 0.25%) cholecalciferol in comparison to the medium (0.55% ± 0.18%) and solvent (0.25% ± 0.16%) controls as well as treatment with 10 ng/mL (1.71% ± 0.60%).

In summary, these findings demonstrate that high-dose cholecalciferol treatments (100 and 1000 ng/mL) cause a significant increase in early and late apoptotic events in comparison to negative controls and low-dose treatments (10 and 40 ng/mL).

**Figure 6:**
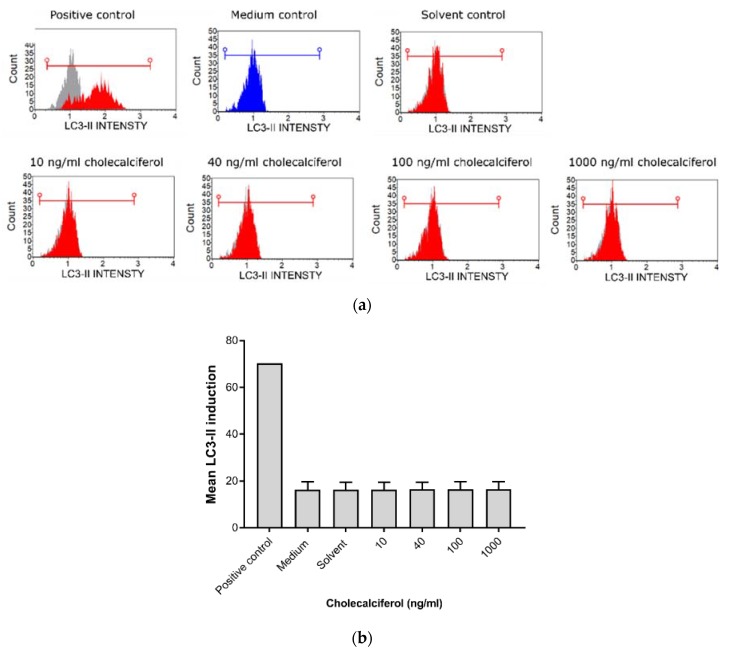
Analysis of autophagic cell death by the Muse™ LC3-II detection assay. (**a**) Histogram of LC3-II intensity in the control and experimental CaSki cultures. (**b**) Statistical analyses of the LC3-II induction assays in the control and experimental CaSki cultures. Cholecalciferol treatments did not cause significant changes in LC3-II expression between the control and experimental cultures. Experiments were performed in triplicate with three biological repeats. Values are expressed as mean ± SEM (*p* < 0.05 was considered statistically significant).

**Figure 7:**
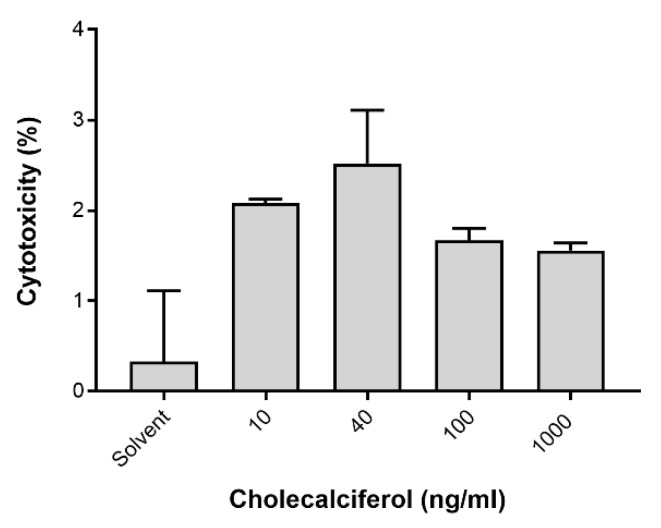
Analysis of necrotic cell death assessed by the lactate dehydrogenase (LDH) assay. Insignificant changes in LDH activity between the control and experimental cultures were identified. Assays were performed on three biological replicates, and each replicate was technically triplicated. Values are expressed as mean ± SEM (*p* < 0.05 was considered statistically significant).

LC3-II was assessed by the Muse™ LC3-II assay (Figure 6) to identify autophagic cell death. LDH was spectrophotometrically quantified to provide a measure of cell membrane damage, which is consistent with necrotic cell death (Figure 7). Neither of the assays identified any significant changes in the induction of these modes of cell death. In addition, the result of 7-AAD necrotic marker analysis (Figure 4e and Figure 5e) are consistent with findings from the LDH assay.

Collectively, the apoptotic biochemical markers can explain the observed decreases in cell count and viability at high-dose treatments.

### 3.4. Morphological Analysis by TEM Showed Features of Apoptosis at High-Dose Cholecalciferol Treatments

**Figure 8:**
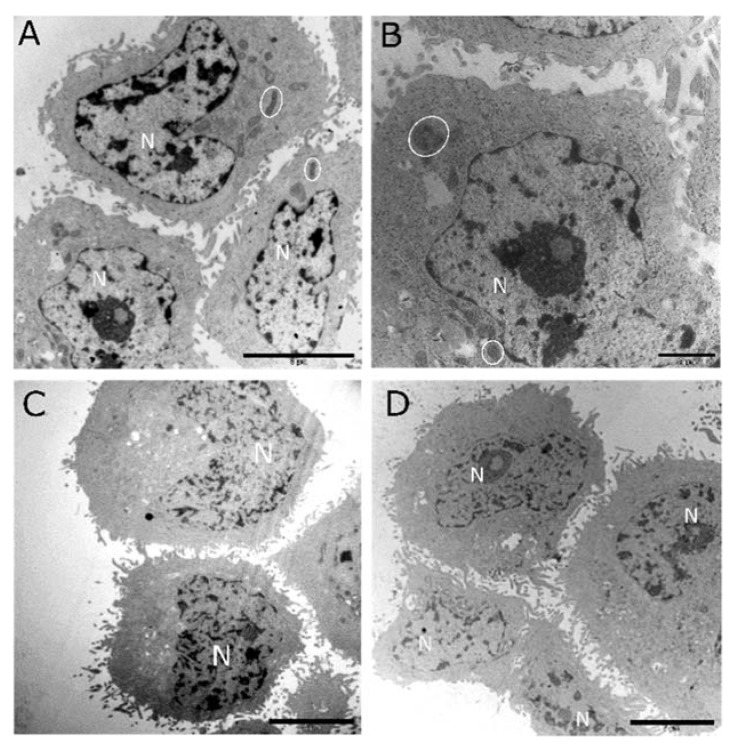
Transmission electron micrographs of the CaSki, control, and low-dose cholecalciferol-treated cultures. Both the medium control (**A**) and solvent control (**B**) showed healthy cells with intact cell and nuclear membranes and clearly defined nuclei (N). Mitochondria (encircled) did not show abnormal morphology. Low-dose cholecalciferol treatments of CaSki cultures of 10 ng/mL (**C**) and 40 ng/mL (**D**) did not show morphological features consistent with cell death. Scale bar: (**A**,**C**) = 5 µm and (**B**,**D**) = 2 µm.

Ultrastructural morphology was investigated using TEM to identify abnormal ultrastructural features of apoptosis, necrosis, and autophagic cell death. The ultrastructural examination supported the biochemical findings regarding apoptotic cell death. Representative micrographs of control cultures (medium shown in Figure 8a and solvent shown in Figure 8b) and cells treated with cholecalciferol at 10 ng/mL (Figure 8c) and 40 ng/mL (Figure 8d) did not show classical features of cell death. Cells demonstrated intact cell membranes with radiating peripheral cellular processes, prominent nuclei with intact nuclear membranes, and numerous mitochondria.

**Figure 9:**
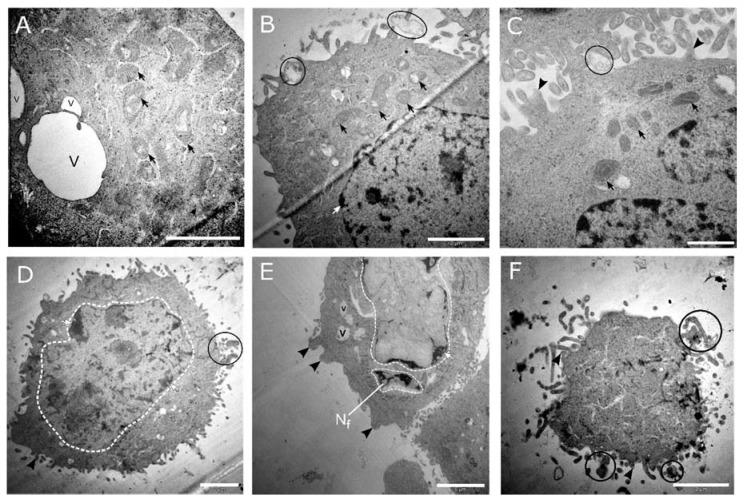
Transmission electron micrographs of CaSki cell cultures treated with 100 and 1000 ng/mL cholecalciferol. The micrographs (**A**–**C**) represent cultures treated with 100 ng/mL cholecalciferol, displaying mitochondria with dilated cristae (black arrows), apoptotic bodies (encircled in black), cell membrane blebbing (black arrowhead), and isolated vacuoles (v). Micrographs (**D**–**F**) represent cultures treated with 1000 ng/mL cholecalciferol, which show features of nuclear apoptosis, including nuclear fragmentation (N_f_) and various stages of karyolysis (encircled white dashed line), membrane budding (black arrowheads), isolated vacuoles (v), and the presence of apoptotic bodies (encircled in black). (**F)** Terminal apoptotic events, demonstrating a shrunken cell with an amorphous cytoplasm surrounded by multiple apoptotic bodies (encircled in black). Scale bars: (**A**,**B**,**D**,**F**) = 2 µm and (**C**) = 1 µm.

High-dose cholecalciferol treatments of 100 ng/mL (Figure 9a–c) and 1000 ng/mL (Figure 9d–f) demonstrated morphological features of apoptosis. Here, cells showed nuclear fragmentation and karyolysis, membrane blebbing, apoptotic bodies, dilated mitochondria, and cell shrinkage.

In summary, apoptotic cell death is supported by significant increase of apoptotic biochemical markers and classical ultrastructural features of apoptosis at high-dose treatments in the CaSki cell line.

## 4. Discussion

The vitamin D compounds (cholecalciferol, calcidiol, and calcitriol) have been shown to exert anticancer actions on various cancer cell lines by mechanisms which include decreased cell proliferation and viability, the induction of apoptosis and autophagic cell death, and decreased metastases and angiogenesis [1,26,27]. In this study, the CaSki cell line, when treated with high doses of cholecalciferol (100 and 1000 ng/mL), showed decreased cell number counts and viability, accompanied by apoptotic cell death as compared to the controls and cultures treated with low doses of cholecalciferol (10 and 40 ng/mL).

The significant decrease in cell count and the statistical increase in the sub-G_1_ fraction of the cell cycle at high-dose cholecalciferol treatments suggests increased cell death in comparison to the controls and low-dose treatments. The inhibition of cell growth has been shown in studies investigating vitamin D action in other cervical cancer cell lines. In HeLa cells, calcitriol has shown time- and dose-dependent decreases in cell proliferation, and the promotion of G_1_ cell cycle arrest [27]. Shruthi et al. also observed 50% growth inhibition in HeLa cells treated with cholecalciferol at a dose of 1250 μM [28]. In addition, calcitriol-treated HeLa and SiHa cells demonstrated decreased dose-dependent cell proliferation [29].

In this study, apoptosis was demonstrated by significant early phosphatidylserine externalization and terminal executioner caspase 3/7 activation at high-dose cholecalciferol treatments. Classical ultrastructural features of apoptosis were also observed at high-dose treatments, which identified nuclear damage, including nuclear fragmentation (karyorrhexis), nuclear lysis (karyolysis), cytoplasmic shrinkage and cell condensation, cell membrane blebbing, and the formation of intact small vesicles (apoptotic cell bodies) [30]. Furthermore, the significant decrease in cell viability was associated with abnormal mitochondrial morphology, which suggests deranged mitochondrial function and associated intrinsic apoptosis [23,30].

This study did not identify a significant occurrence of necrotic and autophagic cell death. Collectively, the LDH cytotoxicity assay, morphological analysis, and flow cytometry studies using the 7-AAD necrosis marker did not indicate significant necrotic cell death. Furthermore, insignificant LC3-II induction and ultrastructural findings did not suggest autophagic cell death.

Although in vitro cell death mechanisms have not been investigated in cervical cancer lines, the observations in this study are consistent with the known pro-apoptotic actions of vitamin D and its metabolites in breast, prostate, colon, gastric, and squamous tumorigenic cell lines [31,32,33]. Studies have shown that vitamin D-induced apoptosis occurs via multiple intracellular mechanisms. Vitamin D-induced apoptosis is mediated by the downregulation of the anti-apoptotic proteins B-cell lymphoma 2 (Bcl-2) and B-cell lymphoma-extra-large (Bcl-XL) [32], and the upregulation of pro-apoptotic proteins Bcl-2-associated X protein (Bax), Bcl2-antagonist/killer 1 (Bak), Bcl-2-associated death promoter (Bad), G_0_-G_1_ switch 2 (GOS2), death-associated protein (DAP-3), Fas-associated death domain (FADD), and caspases [33,34]. Calcitriol has also demonstrated apoptotic induction via the inhibition of the AKT (v-Akt murine thymoma viral oncogene homolog; protein kinase B)-mediated anti-apoptotic signaling pathway, by means of the upregulation of phosphatase and tensin homolog (PTEN) expression in the HGC-27 gastric cancer cell line [35]. In addition, calcitriol has also shown apoptotic initiation in the breast cancer cell line MCF-7 via the regulation of cellular calcium signaling and the recruitment of calcium-dependent apoptotic effectors, such as Ca^2+^-dependent proteases [36]. Moreover, the stimulation of apoptosis by calcitriol in ovarian cancer lines and malignant ovarian tumors led to the induction of microRNA-498, which inhibits mRNA expression of the human telomerase reverse transcriptase, which is essential for cancer growth [37].

In conclusion, this study demonstrates that the high-dose cholecalciferol treatment of the CaSki cell line inhibits cell viability, as demonstrated by reduced cell count, in addition to inducing apoptotic cell death. The local regulation of the VDMS warrants investigation to identify various enzymes and VDRs which may be differentially regulated in order to completely characterize the local metabolism of cholecalciferol in the CaSki cell line. In addition, the mechanisms of apoptotic induction by cholecalciferol in the CaSki cell line require further clarification. This study establishes a promising basis for further research into cholecalciferol and other vitamin D metabolites in the adjunctive treatment of metastatic cervical cancer.
